# [2-Hydroxy-*N*′-(4-oxo-4-phenyl­butan-2-yl­idene)benzohydrazidato(2−)]pyridine­copper(II)

**DOI:** 10.1107/S1600536810047719

**Published:** 2010-11-24

**Authors:** Shu-Ping Zhang, Ying Wei, Si-Chang Shao

**Affiliations:** a Department of Chemistry, Fuyang Normal College, Fuyang, Anhui 236041, People’s Republic of China

## Abstract

The mononuclear title complex, [Cu(C_17_H_14_N_2_O_3_)(C_5_H_5_N)], was synthesized by the reaction of CuCl_2_·2H_2_O with *N*-(4-oxo-4-phenyl­butan-2-yl­idene)benzohydrazide (H_2_ *L*). The central Cu^II^ atom exhibits a distorted square-planar coordination geometry, defined by two O atoms, one N atom from the ligand and one pyridine N atom with Cu—N distances of 1.874 (4) and 1.963 (4) Å, while the Cu—O distances are 1.857 (3) and 1.890 (3) Å. An intra­molecular O—H⋯N inter­action occurs.

## Related literature

For the biological properties of Schiff base–metal complexes, see: Cozzi (2004 ▶). For metallobiomolecules, see: Singh *et al.* (2007 ▶). For metal ions bonded to biologically active compounds, see: Canpolat & Kaya (2004 ▶); Yildiz *et al.* (2004 ▶). For a related structure, see: Shen *et al.* (1997 ▶).
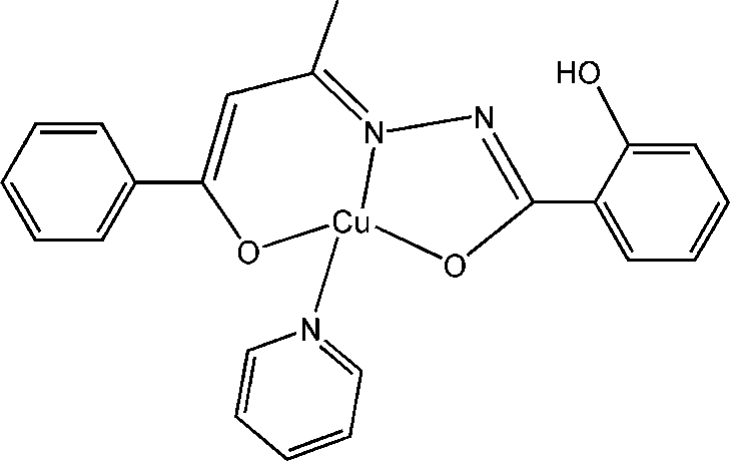


## Experimental

### 

#### Crystal data

[Cu(C_17_H_14_N_2_O_3_)(C_5_H_5_N)]
*M* *_r_* = 436.94
Orthorhombic, 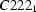

*a* = 7.7096 (8) Å
*b* = 22.906 (2) Å
*c* = 20.983 (2) Å
*V* = 3705.6 (7) Å^3^
*Z* = 8
Mo *K*α radiation
μ = 1.21 mm^−1^
*T* = 298 K
0.28 × 0.20 × 0.20 mm

#### Data collection

Bruker SMART APEXII CCD diffractometer
Absorption correction: multi-scan (*SADABS*; Sheldrick, 1996 ▶) *T*_min_ = 0.728, *T*_max_ = 0.794
13526 measured reflections
4034 independent reflections
3340 reflections with *I* > 2σ(*I*)
*R*_int_ = 0.050

#### Refinement

*R*[*F* ^2^ > 2σ(*F* ^2^)] = 0.057
*wR*(*F* ^2^) = 0.131
*S* = 1.08
4034 reflections
252 parameters
H-atom parameters constrained
Δρ_max_ = 0.45 e Å^−3^
Δρ_min_ = −0.56 e Å^−3^
Absolute structure: Flack (1983 ▶), 1761 Friedel pairs
Flack parameter: 0.08 (3)

### 

Data collection: *APEX2* (Bruker, 2003 ▶); cell refinement: *SAINT* (Bruker, 2003 ▶); data reduction: *SAINT*; program(s) used to solve structure: *SHELXS97* (Sheldrick, 2008 ▶); program(s) used to refine structure: *SHELXL97* (Sheldrick, 2008 ▶); molecular graphics: *SHELXTL* (Sheldrick, 2008 ▶); software used to prepare material for publication: *SHELXTL*.

## Supplementary Material

Crystal structure: contains datablocks global, I. DOI: 10.1107/S1600536810047719/bx2325sup1.cif

Structure factors: contains datablocks I. DOI: 10.1107/S1600536810047719/bx2325Isup2.hkl

Additional supplementary materials:  crystallographic information; 3D view; checkCIF report

## Figures and Tables

**Table 1:** Hydrogen-bond geometry (Å, °)

*D*—H⋯*A*	*D*—H	H⋯*A*	*D*⋯*A*	*D*—H⋯*A*
O1—H1⋯N1	0.82	1.78	2.500 (5)	146

**Fig. 1.**
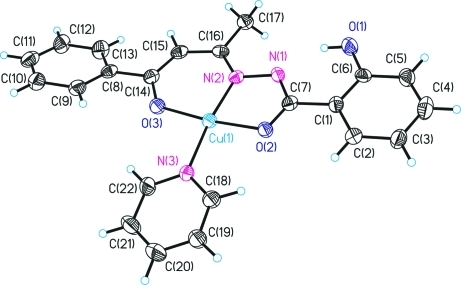
The molecular structure of the title compound, showing 30% probability displacement ellipsoids.

## supplementary crystallographic information

### Comment

Schiff base metal complexes have been widely studied because they have industrial, antifungal, antibacterial, anticancer and herbicidal applications (Cozzi, 2004). It is well known that N atoms play a key role in the coordination of metals at the active sites of numerous metallobiomolecules (Singh, *et al.*, 2007). They serve as models for biological important species and find applications in biomimetic catalytic reactions. Chelating ligands containing N and O donor atoms show broad biological activity and are of special interest because of the variety of ways in which they are bonded to metal ions. It is known that the existence of metal ions bonded to biologically active compounds may enhance their activities (Canpolat, *et al.*, 2004; Yildiz, *et al.*, 2004). Therefore, it is an important study to design and synthesis of new multidentate ligands cotaining N and O atoms and apply to synthesize complexes.

The asymmetric unit is composed of one mononuclear complex, (Fig.1). The central Cu^II^ atom exhibits a distorted square-plannar coordination geometry, defined by two O atoms, one N atom from the ligand molecule and one N atom of the pyridine molecule with Cu—N distances of 1.874 (4) and 1.963 (4) Å while Cu—O distances are 1.857 (3) and 1.890 (3) Å respectively. The Cu—N and Cu—O distances are comparable to those found in other crystallographically characterized Cu^II^ complex (Shen, *et al.* 1997). The crystal structure of the title compound is stabilized by one intramolecular O—H···N interactions with average H···N distances 1.78Å and O—H···N angle 146.3°.

### Experimental

All reagents and solvents were used as obtained commercially without further purification. CuCl_2_.2H_2_O (0.170 mg, 0.1 mmol) was dissolved in 6 ml deionized water, giving a transparent solution, and 1 mL pyridine solution dissolved with *L* (28.2 mg, 0.1 mmol) was dropwised for 0.5 h. After stirring for 8 h, the solution was filtered. Black single crystals of the title compound were obtained from the filtrate after 3 weeks. Analysis calculated (%): C, 60.47; H, 4.38; N, 9.62%; Found: C, 60.15; H, 4.59; N, 9.49%.

### Refinement

H atoms bonded to C atoms were placed geometrically and treated as riding, with C—H distances 0.93–0.96Å and *U*_iso_(H) = 1.2*U*_eq_(C) for the CH while *U*_iso_(H) = 1.5*U*_eq_(C) for the CH_3_ groups. The hydroxyl H atoms were located from difference maps and refined with the O—H distances restrained to 0.82 Å and *U*_iso_(H) = 1.5*U*_eq_(O).

### Crystal data

**Table:**

[Cu(C_17_H_14_N_2_O_3_)(C_5_H_5_N)]	*F*(000) = 1800
*M**_r_* = 436.94	*D*_x_ = 1.566 Mg m^−^^3^
Orthorhombic, *C*222_1_	Mo *K*α radiation, λ = 0.71073 Å
Hall symbol: C 2c 2	Cell parameters from 3542 reflections
*a* = 7.7096 (8) Å	θ = 2.1–23.1°
*b* = 22.906 (2) Å	µ = 1.21 mm^−^^1^
*c* = 20.983 (2) Å	*T* = 298 K
*V* = 3705.6 (7) Å^3^	Block, dark green
*Z* = 8	0.28 × 0.20 × 0.20 mm

### Data collection

**Table:**

Bruker SMART APEXII CCD diffractometer	4034 independent reflections
Radiation source: fine-focus sealed tube	3340 reflections with *I* > 2σ(*I*)
graphite	*R*_int_ = 0.050
phi and ω scans	θ_max_ = 27.0°, θ_min_ = 1.8°
Absorption correction: multi-scan (*SADABS*; Sheldrick, 1996)	*h* = −9→9
*T*_min_ = 0.728, *T*_max_ = 0.794	*k* = −29→27
13526 measured reflections	*l* = −26→26

### Refinement

**Table:**

Refinement on *F*^2^	Secondary atom site location: difference Fourier map
Least-squares matrix: full	Hydrogen site location: inferred from neighbouring sites
*R*[*F*^2^ > 2σ(*F*^2^)] = 0.057	H-atom parameters constrained
*wR*(*F*^2^) = 0.131	*w* = 1/[σ^2^(*F*_o_^2^) + (0.0614*P*)^2^ + 1.8583*P*] where *P* = (*F*_o_^2^ + 2*F*_c_^2^)/3
*S* = 1.08	(Δ/σ)_max_ = 0.003
4034 reflections	Δρ_max_ = 0.45 e Å^−^^3^
252 parameters	Δρ_min_ = −0.56 e Å^−^^3^
0 restraints	Absolute structure: Flack (1983), 1761 Friedel pairs
Primary atom site location: structure-invariant direct methods	Flack parameter: 0.08 (3)

### Special details

**Table:**

Geometry. All e.s.d.'s (except the e.s.d. in the dihedral angle between two l.s. planes) are estimated using the full covariance matrix. The cell e.s.d.'s are taken into account individually in the estimation of e.s.d.'s in distances, angles and torsion angles; correlations between e.s.d.'s in cell parameters are only used when they are defined by crystal symmetry. An approximate (isotropic) treatment of cell e.s.d.'s is used for estimating e.s.d.'s involving l.s. planes.
Refinement. Refinement of *F*^2^ against ALL reflections. The weighted *R*-factor *wR* and goodness of fit *S* are based on *F*^2^, conventional *R*-factors *R* are based on *F*, with *F* set to zero for negative *F*^2^. The threshold expression of *F*^2^ > σ(*F*^2^) is used only for calculating *R*-factors(gt) *etc*. and is not relevant to the choice of reflections for refinement. *R*-factors based on *F*^2^ are statistically about twice as large as those based on *F*, and *R*- factors based on ALL data will be even larger.

### Fractional atomic coordinates and isotropic or equivalent isotropic displacement parameters (Å^2^)

**Table:**

	*x*	*y*	*z*	*U*_iso_*/*U*_eq_	
Cu1	0.26164 (7)	0.56424 (2)	0.75389 (2)	0.03955 (17)	
C1	0.4395 (6)	0.4324 (2)	0.8613 (2)	0.0396 (11)	
C2	0.5248 (7)	0.4551 (2)	0.9121 (2)	0.0496 (13)	
H2	0.5322	0.4955	0.9165	0.059*	
C3	0.5994 (7)	0.4205 (3)	0.9567 (2)	0.0558 (14)	
H3	0.6569	0.4370	0.9913	0.067*	
C4	0.5895 (8)	0.3611 (3)	0.9504 (3)	0.0653 (17)	
H4	0.6401	0.3369	0.9807	0.078*	
C5	0.5071 (9)	0.3384 (2)	0.9008 (2)	0.0534 (12)	
H5	0.5021	0.2980	0.8964	0.064*	
C6	0.4294 (7)	0.3728 (2)	0.8558 (2)	0.0440 (12)	
C7	0.3627 (6)	0.4715 (2)	0.8143 (2)	0.0386 (11)	
C8	0.0179 (5)	0.60424 (13)	0.58645 (12)	0.0411 (11)	
C9	0.0827 (5)	0.66043 (14)	0.57885 (14)	0.0524 (13)	
H9A	0.1631	0.6751	0.6078	0.063*	
C10	0.0275 (5)	0.69465 (12)	0.52805 (16)	0.0589 (15)	
H10A	0.0709	0.7322	0.5230	0.071*	
C11	−0.0925 (5)	0.67268 (15)	0.48484 (14)	0.0617 (16)	
H11A	−0.1295	0.6956	0.4509	0.074*	
C12	−0.1574 (4)	0.61649 (16)	0.49245 (15)	0.0614 (15)	
H12A	−0.2377	0.6018	0.4635	0.074*	
C13	−0.1022 (5)	0.58227 (12)	0.54325 (16)	0.0589 (15)	
H13A	−0.1456	0.5447	0.5483	0.071*	
C14	0.0822 (6)	0.5682 (2)	0.6383 (2)	0.0423 (11)	
C15	0.0672 (6)	0.5104 (2)	0.6359 (2)	0.0435 (12)	
H15	0.0074	0.4954	0.6011	0.052*	
C16	0.1308 (6)	0.4698 (2)	0.6795 (2)	0.0391 (11)	
C17	0.1033 (7)	0.4076 (2)	0.6673 (2)	0.0476 (12)	
H17A	0.0384	0.3908	0.7017	0.071*	
H17B	0.2134	0.3883	0.6638	0.071*	
H17C	0.0399	0.4029	0.6282	0.071*	
C18	0.3924 (7)	0.6464 (2)	0.8448 (2)	0.0532 (14)	
H18	0.4366	0.6128	0.8636	0.064*	
C19	0.4202 (9)	0.6974 (3)	0.8736 (3)	0.0635 (17)	
H19	0.4847	0.6989	0.9110	0.076*	
C20	0.3560 (11)	0.7459 (3)	0.8488 (3)	0.080 (2)	
H20	0.3752	0.7818	0.8682	0.096*	
C21	0.2630 (12)	0.7419 (2)	0.7953 (3)	0.085 (2)	
H21	0.2139	0.7750	0.7771	0.102*	
C22	0.2413 (9)	0.6887 (2)	0.7680 (2)	0.0658 (17)	
H22	0.1769	0.6862	0.7307	0.079*	
N1	0.2780 (5)	0.44657 (14)	0.76881 (16)	0.0376 (8)	
N2	0.2134 (4)	0.48748 (16)	0.72866 (15)	0.0353 (8)	
N3	0.3066 (5)	0.64115 (17)	0.79176 (17)	0.0400 (9)	
O1	0.3475 (6)	0.34649 (15)	0.80904 (17)	0.0602 (10)	
H1	0.3070	0.3710	0.7848	0.090*	
O2	0.3808 (5)	0.52533 (13)	0.82017 (15)	0.0428 (8)	
O3	0.1558 (5)	0.59686 (14)	0.68281 (15)	0.0499 (9)	

### Atomic displacement parameters (Å^2^)

**Table:**

	*U*^11^	*U*^22^	*U*^33^	*U*^12^	*U*^13^	*U*^23^
Cu1	0.0412 (3)	0.0412 (3)	0.0363 (3)	0.0042 (3)	−0.0104 (3)	−0.0039 (2)
C1	0.034 (2)	0.045 (3)	0.039 (2)	0.004 (2)	0.0083 (18)	0.008 (2)
C2	0.058 (4)	0.050 (3)	0.041 (3)	0.005 (3)	0.000 (2)	−0.002 (2)
C3	0.059 (4)	0.066 (4)	0.043 (3)	0.008 (3)	−0.006 (2)	0.007 (2)
C4	0.056 (4)	0.085 (5)	0.055 (3)	0.014 (4)	0.005 (3)	0.027 (3)
C5	0.058 (3)	0.050 (3)	0.052 (3)	0.007 (3)	0.010 (3)	0.011 (2)
C6	0.050 (3)	0.041 (3)	0.041 (3)	0.006 (2)	0.014 (2)	−0.003 (2)
C7	0.030 (2)	0.050 (3)	0.036 (2)	0.002 (2)	−0.004 (2)	−0.004 (2)
C8	0.035 (3)	0.058 (3)	0.031 (2)	0.005 (3)	−0.005 (2)	−0.008 (2)
C9	0.060 (3)	0.056 (3)	0.041 (3)	0.003 (3)	−0.004 (2)	−0.005 (2)
C10	0.064 (4)	0.061 (3)	0.052 (3)	0.005 (3)	−0.001 (3)	0.003 (3)
C11	0.064 (4)	0.082 (5)	0.039 (3)	0.018 (3)	−0.002 (3)	−0.002 (3)
C12	0.058 (3)	0.075 (4)	0.051 (3)	0.007 (3)	−0.018 (3)	0.007 (3)
C13	0.049 (3)	0.074 (4)	0.054 (3)	0.006 (3)	−0.021 (3)	0.003 (3)
C14	0.034 (2)	0.056 (3)	0.036 (2)	0.012 (3)	−0.0044 (19)	−0.003 (2)
C15	0.040 (3)	0.057 (3)	0.034 (2)	−0.002 (2)	−0.008 (2)	−0.013 (2)
C16	0.032 (2)	0.042 (3)	0.043 (2)	−0.002 (2)	0.007 (2)	−0.009 (2)
C17	0.050 (3)	0.047 (3)	0.046 (3)	−0.004 (3)	−0.011 (2)	−0.008 (2)
C18	0.061 (4)	0.051 (3)	0.048 (3)	0.003 (3)	−0.019 (3)	−0.007 (2)
C19	0.084 (4)	0.049 (3)	0.057 (3)	0.007 (3)	−0.026 (3)	−0.006 (3)
C20	0.124 (7)	0.044 (3)	0.072 (4)	0.005 (4)	−0.018 (4)	−0.013 (3)
C21	0.133 (7)	0.045 (3)	0.078 (4)	0.028 (4)	−0.036 (5)	−0.012 (3)
C22	0.097 (5)	0.053 (3)	0.048 (3)	0.014 (4)	−0.028 (4)	0.002 (2)
N1	0.038 (2)	0.0335 (19)	0.0408 (17)	0.0034 (16)	0.0008 (17)	0.0041 (14)
N2	0.0283 (19)	0.045 (2)	0.0327 (16)	0.0030 (16)	0.0005 (15)	−0.0042 (15)
N3	0.043 (2)	0.042 (2)	0.0355 (18)	0.0042 (18)	−0.0054 (16)	0.0018 (17)
O1	0.081 (3)	0.040 (2)	0.059 (2)	0.003 (2)	−0.015 (2)	−0.0024 (18)
O2	0.051 (2)	0.0356 (19)	0.0417 (17)	0.0018 (15)	−0.0138 (16)	−0.0037 (14)
O3	0.064 (2)	0.044 (2)	0.0417 (17)	0.0059 (18)	−0.0178 (16)	−0.0048 (16)

### Geometric parameters (Å, °)

**Table:**

Cu1—O3	1.857 (3)	C11—H11A	0.9300
Cu1—N2	1.874 (4)	C12—C13	1.3900
Cu1—O2	1.890 (3)	C12—H12A	0.9300
Cu1—N3	1.963 (4)	C13—H13A	0.9300
C1—C2	1.357 (7)	C14—O3	1.276 (5)
C1—C6	1.373 (7)	C14—C15	1.328 (7)
C1—C7	1.457 (7)	C15—C16	1.394 (7)
C2—C3	1.355 (7)	C15—H15	0.9300
C2—H2	0.9300	C16—N2	1.278 (6)
C3—C4	1.369 (9)	C16—C17	1.462 (7)
C3—H3	0.9300	C17—H17A	0.9600
C4—C5	1.327 (8)	C17—H17B	0.9600
C4—H4	0.9300	C17—H17C	0.9600
C5—C6	1.367 (7)	C18—N3	1.300 (6)
C5—H5	0.9300	C18—C19	1.332 (8)
C6—O1	1.314 (6)	C18—H18	0.9300
C7—O2	1.247 (5)	C19—C20	1.323 (8)
C7—N1	1.291 (6)	C19—H19	0.9300
C8—C9	1.3900	C20—C21	1.335 (9)
C8—C13	1.3900	C20—H20	0.9300
C8—C14	1.453 (5)	C21—C22	1.356 (7)
C9—C10	1.3900	C21—H21	0.9300
C9—H9A	0.9300	C22—N3	1.300 (6)
C10—C11	1.3900	C22—H22	0.9300
C10—H10A	0.9300	N1—N2	1.355 (5)
C11—C12	1.3900	O1—H1	0.8200
			
O3—Cu1—N2	93.64 (15)	C12—C13—H13A	120.0
O3—Cu1—O2	173.85 (15)	C8—C13—H13A	120.0
N2—Cu1—O2	82.05 (14)	O3—C14—C15	125.4 (4)
O3—Cu1—N3	92.39 (15)	O3—C14—C8	114.0 (4)
N2—Cu1—N3	172.50 (15)	C15—C14—C8	120.6 (4)
O2—Cu1—N3	92.27 (14)	C14—C15—C16	127.5 (4)
C2—C1—C6	118.3 (5)	C14—C15—H15	116.2
C2—C1—C7	119.5 (5)	C16—C15—H15	116.2
C6—C1—C7	122.1 (5)	N2—C16—C15	119.6 (4)
C3—C2—C1	121.6 (5)	N2—C16—C17	121.5 (4)
C3—C2—H2	119.2	C15—C16—C17	118.9 (4)
C1—C2—H2	119.2	C16—C17—H17A	109.5
C2—C3—C4	119.4 (5)	C16—C17—H17B	109.5
C2—C3—H3	120.3	H17A—C17—H17B	109.5
C4—C3—H3	120.3	C16—C17—H17C	109.5
C5—C4—C3	119.5 (5)	H17A—C17—H17C	109.5
C5—C4—H4	120.2	H17B—C17—H17C	109.5
C3—C4—H4	120.2	N3—C18—C19	123.4 (5)
C4—C5—C6	121.7 (5)	N3—C18—H18	118.3
C4—C5—H5	119.2	C19—C18—H18	118.3
C6—C5—H5	119.2	C20—C19—C18	119.9 (6)
O1—C6—C5	117.5 (5)	C20—C19—H19	120.0
O1—C6—C1	123.1 (5)	C18—C19—H19	120.0
C5—C6—C1	119.4 (5)	C19—C20—C21	118.3 (6)
O2—C7—N1	124.5 (4)	C19—C20—H20	120.9
O2—C7—C1	119.7 (4)	C21—C20—H20	120.9
N1—C7—C1	115.7 (4)	C20—C21—C22	118.9 (6)
C9—C8—C13	120.0	C20—C21—H21	120.6
C9—C8—C14	119.3 (3)	C22—C21—H21	120.6
C13—C8—C14	120.6 (3)	N3—C22—C21	122.9 (5)
C10—C9—C8	120.0	N3—C22—H22	118.5
C10—C9—H9A	120.0	C21—C22—H22	118.5
C8—C9—H9A	120.0	C7—N1—N2	109.9 (4)
C9—C10—C11	120.0	C16—N2—N1	117.8 (4)
C9—C10—H10A	120.0	C16—N2—Cu1	128.6 (3)
C11—C10—H10A	120.0	N1—N2—Cu1	113.6 (3)
C12—C11—C10	120.0	C22—N3—C18	116.6 (4)
C12—C11—H11A	120.0	C22—N3—Cu1	122.0 (3)
C10—C11—H11A	120.0	C18—N3—Cu1	121.2 (4)
C11—C12—C13	120.0	C6—O1—H1	109.5
C11—C12—H12A	120.0	C7—O2—Cu1	109.9 (3)
C13—C12—H12A	120.0	C14—O3—Cu1	125.2 (3)
C12—C13—C8	120.0		
			
C6—C1—C2—C3	0.2 (8)	N3—C18—C19—C20	1.4 (10)
C7—C1—C2—C3	−179.5 (5)	C18—C19—C20—C21	0.5 (12)
C1—C2—C3—C4	0.2 (9)	C19—C20—C21—C22	−1.4 (13)
C2—C3—C4—C5	0.1 (9)	C20—C21—C22—N3	0.3 (13)
C3—C4—C5—C6	−0.9 (10)	O2—C7—N1—N2	0.3 (6)
C4—C5—C6—O1	−178.5 (5)	C1—C7—N1—N2	180.0 (3)
C4—C5—C6—C1	1.3 (9)	C15—C16—N2—N1	−177.9 (4)
C2—C1—C6—O1	178.9 (5)	C17—C16—N2—N1	1.1 (6)
C7—C1—C6—O1	−1.5 (8)	C15—C16—N2—Cu1	1.6 (6)
C2—C1—C6—C5	−1.0 (7)	C17—C16—N2—Cu1	−179.4 (4)
C7—C1—C6—C5	178.7 (5)	C7—N1—N2—C16	177.6 (4)
C2—C1—C7—O2	2.3 (7)	C7—N1—N2—Cu1	−1.9 (4)
C6—C1—C7—O2	−177.4 (5)	O3—Cu1—N2—C16	−1.8 (4)
C2—C1—C7—N1	−177.4 (4)	O2—Cu1—N2—C16	−177.4 (4)
C6—C1—C7—N1	3.0 (7)	O3—Cu1—N2—N1	177.7 (3)
C13—C8—C9—C10	0.0	O2—Cu1—N2—N1	2.1 (3)
C14—C8—C9—C10	177.5 (4)	C21—C22—N3—C18	1.5 (10)
C8—C9—C10—C11	0.0	C21—C22—N3—Cu1	176.4 (6)
C9—C10—C11—C12	0.0	C19—C18—N3—C22	−2.4 (8)
C10—C11—C12—C13	0.0	C19—C18—N3—Cu1	−177.3 (5)
C11—C12—C13—C8	0.0	O2—Cu1—N3—C22	−175.0 (5)
C9—C8—C13—C12	0.0	O3—Cu1—N3—C18	−176.3 (4)
C14—C8—C13—C12	−177.4 (4)	O2—Cu1—N3—C18	−0.3 (4)
C9—C8—C14—O3	19.2 (5)	N1—C7—O2—Cu1	1.4 (6)
C13—C8—C14—O3	−163.4 (3)	C1—C7—O2—Cu1	−178.3 (3)
C9—C8—C14—C15	−158.8 (4)	N2—Cu1—O2—C7	−1.8 (3)
C13—C8—C14—C15	18.7 (6)	N3—Cu1—O2—C7	173.3 (4)
O3—C14—C15—C16	−1.8 (9)	C15—C14—O3—Cu1	1.0 (7)
C8—C14—C15—C16	175.9 (4)	C8—C14—O3—Cu1	−176.8 (3)
C14—C15—C16—N2	0.4 (8)	N2—Cu1—O3—C14	0.5 (4)
C14—C15—C16—C17	−178.7 (5)	N3—Cu1—O3—C14	−175.0 (4)

### Hydrogen-bond geometry (Å, °)

**Table:**

*D*—H···*A*	*D*—H	H···*A*	*D*···*A*	*D*—H···*A*
O1—H1···N1	0.82	1.78	2.500 (5)	146
